# Association between functional combined anteversion and dislocation after revision total hip arthroplasty

**DOI:** 10.1186/s42836-026-00383-w

**Published:** 2026-04-10

**Authors:** Yuta Hieda, Hyonmin Choe, Koki Abe, Hiroyuki Ike, Masashi Shimoda, Hironori Yamane, Akira Morita, Kosuke Sumi, Ken Kumagai, Naomi Kobayashi, Yutaka Inaba

**Affiliations:** 1 Department of Orthopedic Surgery, Yokohama City University, 3-9 Fukuura, Kanazawa-Ku, Yokohama, Kanagawa 236-0004 Japan; 2 Department of Orthopedic Surgery, Yokohama City University Medical Center, 4-57 Urafune, Minami-Ku, Yokohama, Kanagawa 232-0024 Japan

**Keywords:** Dislocation, Femoral rotation, Revision total hip arthroplasty, Combined anteversion

## Abstract

**Background:**

Dislocation is a serious complication that should be avoided in total hip arthroplasty (THA). Combined anteversion (CA) of the cup and stem is a concept for appropriate implant positioning; however, the effect of functional changes in femoral rotation has not been well investigated. In this study, we investigated whether functional CA, considering femoral rotation, is associated with dislocation in patients who underwent revision THA.

**Methods:**

Overall, 82 patients who underwent revision THA and had at least one year of follow-up with pre-operative and post-operative supine computed tomography imaging were included. The cup and stem were placed with a target combined angle of 37.3° using Widmer’s formula. Anatomical and functional CAs were calculated post-operatively. Functional CA was defined as the sum of cup anteversion and stem anteversion, with femoral external rotation. Patient demographics, component alignment parameters, CA, and their association with post-operative dislocation were statistically evaluated.

**Results:**

Dislocation was observed in 12 patients. In these dislocated cases, there were no significant differences in cup angle, stem angle, and anatomical CA compared to non-dislocated cases. However, dislocated cases showed significantly higher values of functional CA (50.0 ± 17.4° [range, 5.5–67.6] vs. 35.6 ± 13.0° [range, 4.0–68.8], *p* = 0.022) and significant deviation from identical CA [15.0 ± 8.9° [range, 3.1–31.8] vs. 7.5 ± 8.1° [range, 0.1–33.3], *p* = 0.014).

**Conclusions:**

Functional CA, considering femoral rotation, was associated with post-operative dislocation after revision THA. Therefore, consideration of femoral rotation may be important for implant positioning in revision THA.

**Supplementary Information:**

The online version contains supplementary material available at 10.1186/s42836-026-00383-w.

## Introduction

Dislocation is a major complication of total hip arthroplasty (THA), and its incidence is even higher after revision THA. The reported dislocation rate after revision THA ranges from 4–30%, and additional revision surgery may be required in some cases [1–4]. Therefore, preventing post-operative dislocation is essential during pre-operative planning. One reason for dislocation is inappropriate implant placement, which causes implant impingement and instability of the hip joint [5–7]. The combined anteversion (CA) angle is the sum of the acetabular and femoral anteversion angles and is crucial for minimizing the dislocation risk [8–10]. Higher CA increases the risk of posterior impingement and anterior dislocation [11].

As CA changes functionally with changes in pelvic tilt and femoral rotation [12–14], external femoral rotation may be a risk factor for dislocation after revision THA [15]. Combined anteversion (CA) planning is typically based on anatomical CA (ACA), which may differ from functional CA (FCA). However, no study has evaluated the relationship between dislocation after revision THA and the effect of femoral rotation on FCA. Therefore, the purpose of this study was to quantify the difference between ACA and FCA using computed tomography (CT) and to evaluate the association between FCA and post-operative dislocation after revision THA.

## Materials and methods

### Ethics approval and informed consent statement

This retrospective study was approved by our institutional review board of Yokohama City University (approval number: B241205073). Informed consent was obtained through passive consent via a notice on the website. All methods were conducted in accordance with applicable guidelines and regulations. Participants were allowed to decline participation through public notification.

### Participants

**Fig. 1:**
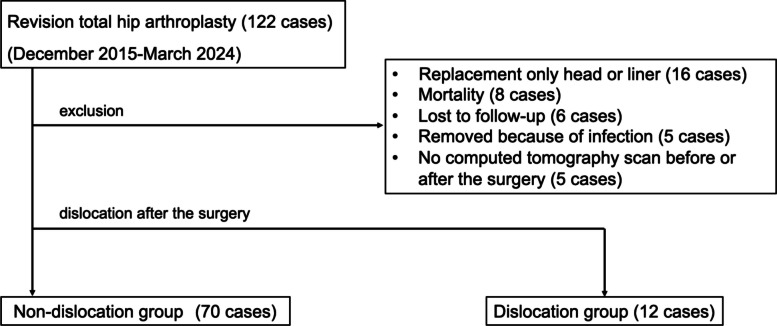
Flowchart of the patient inclusion criteria

We screened patients who underwent revision THA at our institution from December 2015 to March 2024. The exclusion criteria were as follows: head or linear replacement only (*n* = 16), post-operative mortality for any reason (*n* = 8), loss to follow-up within one year (*n* = 6), implant removal because of uncontrolled infection (*n* = 5), and missing pre-operative or post-operative CT imaging data (*n* = 5). We enrolled 82 of 122 patients who underwent revision THA at our institution (Fig. 1). Patients who experienced dislocation at least once after revision THA were included in the dislocation group, whereas those who did not were included in the non-dislocation group.

### Data collection

Data on patient background [sex, age, body mass index (BMI), follow-up period, number of revision surgeries, presence of peri-prosthetic joint infection, functional pelvic plane (FPP) angle in the supine position, and external femoral rotation angle before one-stage or two-stage revision surgery], surgical factors [approach at revision (anterior, lateral, posterior), two-stage surgery, use of reconstructive cages and dual mobility cup, use of cemented or cementless cup and stem, anatomical stem anteversion angle, cup anteversion angle, and inclination], and postoperative factors (external femoral rotation angle, leg length difference, and pelvic, femoral and total offset difference) were collected retrospectively from the medical records. All angles, except for post-operative, the angles of femoral rotation and stem anteversion, were calculated in the anatomical pelvic plane (APP) and FPP in both groups.

For all patients, the safe zone was set as 40 ± 10° for cup inclination and 37.3 ± 10° for ACA or FCA, and scatter plots were used to investigate cases that deviated from the safe zone [9, 10]. Additionally, for all patients in the dislocation group, we investigated the diagnosis, direction of the dislocation, duration from revision surgery to dislocation, ACA and FCA, and the difference between both in the FPP, and the outcome after the dislocation.

### Measurement of angles

**Fig. 2:**
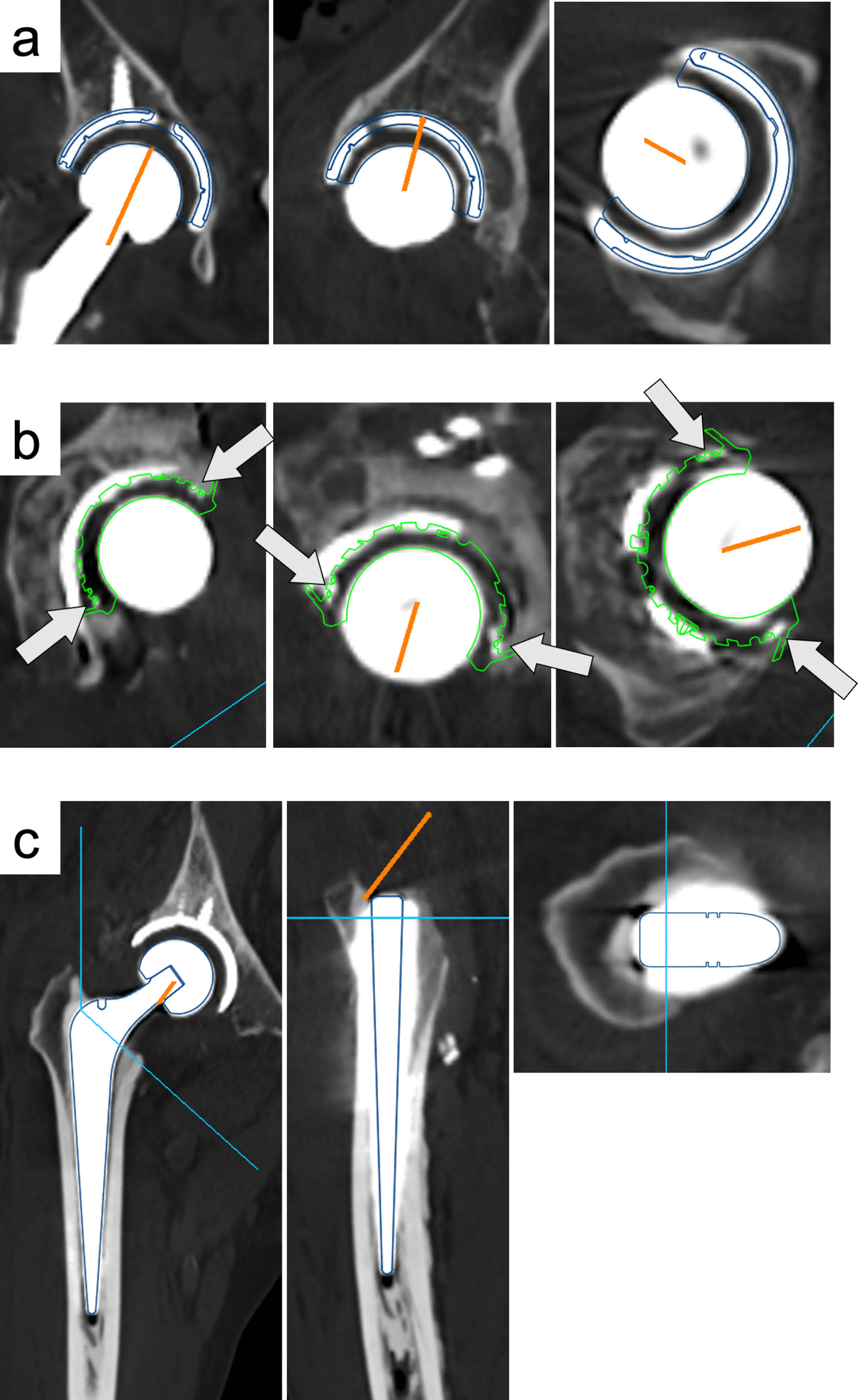
The figure shows how the angle of cup component placement is measured using three-dimensional analysis software. (**a**) For cementless cups or metal cemented cups, same-sized components are placed in three directions and adapted on computed tomography (CT) images within 2 weeks after surgery. (**b**) For polyethylene cemented cups, the same-sized components are adapted on CT images within 2 weeks after surgery, using the wire placed around the rim of the polyester cup as an indicator. The arrows point to the wire. (**c**) For cementless or cemented stems, same-sized components are placed in three directions and adapted on CT images

All patients underwent CT (Sensation 16; Siemens AG, Erlangen, Germany; 120 kV and 300 mA, 1.5-mm slice) at a height from the superior aspect of the first vertebral body to below the knee bilaterally at 1 month before and within 2 weeks after surgery. Three-dimensional modelling of the pelvis and its components was performed using CT images in an analysis software (ZedHip; Lexi Co., Ltd., Tokyo, Japan) (Fig. 2).

In angle measurements, both the APP and the FPP were used as reference planes. The APP was defined by the bilateral anterior superior iliac spines and the pubic symphysis, whereas the FPP was defined as the pelvic orientation in the supine position on the CT table using three-dimensional modelling of the pelvis, considering functional pelvic alignment. ACA was defined as the sum of cup anteversion and anatomical stem anteversion. FCA was defined as the sum of cup anteversion and functional stem anteversion.

To evaluate component alignment, CA was assessed according to Widmer’s formula: stem anteversion × 0.7 + cup anteversion angle [9]. In this formula, anatomical stem anteversion was used for ACA, and functional stem anteversion, which was defined as anatomical stem anteversion adjusted for femoral external rotation, was used for FCA. The absolute difference between the ideal value of 37.3°, according to Widmer’s study, and ACA or FCA was calculated [9]. Detailed descriptions of the measurement methods are provided in the supplementary materials. All measurements of angles and implants were independently performed by two experienced hip surgeons. Interobserver reliability was assessed using intraclass correlation coefficients (ICC). The mean value of the two measurements was used for statistical analysis to minimize interobserver variability.

### Data analysis

Univariate analysis was performed for all factors, and multivariate analysis was performed only for those showing significant differences in the univariate analysis. Since functional stem anteversion and FCA represent the same alignment construct, they were not included simultaneously in the multivariate analysis. Instead, the absolute deviation from the ideal CA (|37.3° − FCA|) was used as the representative measure of CA. Variables divided by one- or two-stage revision were not included in the multivariate analysis. Differences between the dislocation and non-dislocation groups were evaluated using the Wilcoxon rank-sum test or Fisher’s exact test. Statistical significance was set at *p* < 0.05*.* Power calculations or sample size calculations could not be performed in advance due to the limited number of dislocation events and the retrospective study design. Therefore, multivariate analysis was performed exploratively to evaluate factors potentially associated with post-operative dislocation. Multivariate analysis was performed using the forced entry method for post-operative dislocation, and a forward–backward stepwise procedure was additionally applied to confirm the stability of the final model. Statistical analysis was performed using JMP Pro version 17.0 (SAS Institute, Inc., Cary, NC, USA) for the univariate analysis and SPSS Statistics (IBM Corp., Armonk, NY, USA) for the multivariate analysis.

## Results

Among 82 patients who underwent revision THA, 12 experienced dislocations (10 hips with anterior dislocation, 2 hips with posterior dislocation), whereas 70 never experienced dislocation. No significant differences in sex, age, BMI, or follow-up period were found between the two groups (Table 1). Six cases performed re-revision surgery, with three cases in each group. In each group, one case underwent two-stage revision surgery twice for peri-prosthetic joint infection, and two cases underwent re-revision for aseptic loosening. The number of patients diagnosed with peri-prosthetic infections did not differ significantly between the groups. The FPP was significantly lower and more posteriorly tilted in the dislocated group than in the non-dislocation group (− 6 ± 7.3° [range, − 25° to 4°] vs. − 1.5 ± 8.0° [range, − 28° to 14°], *p* = 0.036) The preoperative external femoral rotation angle did not differ significantly before one-stage surgery, whereas before two-stage surgery, the angle was higher in the dislocation group than in the non-dislocation group. The surgical approach was the lateral approach in all but one case, for which the posterior approach was used, in the non-dislocation group. Although no significant difference in periprosthetic pre-operative peri-prosthetic joint infection was observed between the two groups, the number of patients who underwent two-stage revision surgery was significantly higher in the dislocation group (9 hips [75.0%] vs. 22 hips [31.4%], *p* = 0.008). Among the components, only the cemented stem was used significantly more in the dislocation group (11 hips [91.7%] vs. 36 hips [51.4%], *p* = 0.010). No significant difference in anatomical stem anteversion, cup placement angle (inclination and anteversion) in the APP or FPP, post-operative external femoral rotation angle, leg length difference, or offset difference was noted. Interobserver reliability for the angle measurements was good to excellent (ICC = 0.75 − 0.98). However, the functional stem anteversion angle was significantly higher in the dislocation group than in the non-dislocation group, likely because of femoral rotation (44 ± 20.0° [range, − 16° to 59°] vs. 31 ± 15.6° [range, − 14° to 68°], *p* = 0.021). Although ACA was not significantly different between the groups in the APP or FPP, FCA, considering the femoral rotation in the only FPP was significantly higher in the dislocation group (50.0 ± 17.4° [range, 5.5° to 67.6°] vs. 35.6 ± 13.0° [range, 4.0° to 68.8°], *p* = 0.022). In the deviation from Widmer’s ideal value of 37.3°, only the absolute difference with FCA in the FPP was significantly higher in the dislocation group than in the non-dislocation group (15.0 ± 8.9° [range, 3.1° to 31.8°] vs. 7.5 ± 8.1° [range, 0.1° to 33.3°], *p* = 0.014) [9].

**Table 1:** Univariate analysis of the dislocation and non-dislocation groups using the Wilcoxon rank sum test or Fisher’s exact test

		**Dislocation** **(*n* = 12)**	**Non-Dislocation** **(*n* = 70)**	***p*-value** **(95% CI)**
Female sex, *n* (%)		6 (50.0)	41 (58.6)	0.579
Age (years)		71.5 ± 8.6 (55 to 84)	71.0 ± 11.8 (32 to 92)	0.462
Body mass index (kg/m^2^)		22.3 ± 5.4 (18 to 39)	21.8 ± 4.2 (15 to 34)	0.963
Follow-up period (months)		45.0 ± 24.5 (12 to 94)	52.5 ± 27.8 (12 to 111)	0.948
Number of revision surgeries, *n* (%)	once	9 (75.0)	67 (95.0)	
	twice	3 (25.0)	3 (4.9)	
Periprosthetic joint infection, *n* (%)		8 (66.7)	24 (34.3)	0.053
FPP angle in supine position (°)		− 6 ± 7.3 (− 25 to 4)	− 1.5 ± 8.0 (− 28 to 14)	0.036*
Preoperative external femoral rotation (°)	one-stage	11.5 ± 33.1 (− 2 to 61) (*n* = 3)	3.3 ± 21.5 (− 26 to 61) (*n* = 48)	0.378
	two-stage	42.5 ± 24.6 (3 to 93) (*n* = 9)	24.8 ± 25.3 (− 38 to 77) (*n* = 22)	0.064
Approach, *n* (%)	Anterior	0 (0.0)	0 (0.0)	
	Lateral	12 (100)	69 (98.6)	
	Posterior	0 (0.0)	1 (1.4)	
Two-stage revision, *n* (%)		9 (75.0)	22 (31.4)	0.008**
Reconstruction cage, *n* (%)		9 (75.0)	30 (42.9)	0.060
Dual mobility, *n* (%)		2 (16.7)	15 (21.4)	0.707
Cement, *n* (%)	Cup	9 (75.0)	32 (45.7)	0.116
	Stem	11 (91.7)	36 (51.4)	0.010*
Anatomical stem anteversion (°)		26.3 ± 17.5 (− 10 to 45)	24.5 ± 12.3 (− 5 to 57)	0.906
Cup anteversion (°)	APP	13.4 ± 4.5 (7.7 to 25.5)	15.2 ± 9.6 (− 14.6 to 36.2)	0.637
	FPP	17.6 ± 8.3 (5 to 37)	17.3 ± 8.5 (− 5 to 40)	0.401
Cup inclination (°)	APP	38.0 ± 7.5 (25.1 to 51.5)	37.8 ± 7.0 (24.2 to 53.5)	0.844
	FPP	40.9 ± 7.4 (26 to 51)	38.7 ± 6.7 (25 to 55)	0.499
Postoperative external femoral rotation (°)		9.8 ± 15.6 (− 6 to 40)	5.3 ± 13.0 (− 24 to 33)	0.087
Leg length difference (mm)	APP	− 5.8 ± 16.3 (− 47.9 to 12.9)	− 2.7 ± 15.7 (− 39.9 to 47.9)	0.689
	FPP	− 4.2 ± 16.6 (− 49.0 to 13.1)	− 2.6 ± 15.6 (− 37.9 to 49.0)	0.839
Pelvic offset difference (mm)		− 1.7 ± 6.8 (− 12.9 to 7.0)	− 3.2 ± 8.5 (− 24.3 to 19.3)	0.753
Femoral offset difference (mm)		4.5 ± 9.8 (− 14.8 to 20.6)	0.9 ± 9.6 (− 16.5 to 28.8)	0.937
Total offset difference (mm)		1.0 ± 8.7 (− 13.9 to 14.2)	− 0.1 ± 11.3 (− 36.8 to 35.2)	0.943
Functional stem anteversion (°)		44 ± 20.0 (− 16 to 59)	31 ± 15.6 (− 14 to 68)	0.021*
ACA (°)	APP	28.8 ± 13.7 (6.1 to 55.3)	32.4 ± 11.0 (10.7 to 56.5)	0.773
	FPP	37.1 ± 13.7 (9.7 to 56.5)	33.7 ± 10.4 (9.8 to 60.1)	0.550
FCA (°)	APP	45.7 ± 15.1 (1.9 to 56.2)	35.5 ± 13.3 (− 2.9 to 75.2)	0.063
	FPP	50.0 ± 17.4 (5.5 to 67.6)	35.6 ± 13.0 (4.0 to 68.8)	0.022*
|37.3° − ACA|	APP	9.6 ± 8.7 (1.7 to 31.2)	8.7 ± 7.2 (0.4 to 26.6)	0.383
	FPP	6.9 ± 9.7 (0.3 to 27.6)	7.1 ± 7.1 (0.0 to 27.5)	0.748
|37.3° − FCA|	APP	10.2 ± 8.4 (3.3 to 35.4)	8.1 ± 8.5 (0.0 to 40.2)	0.179
	FPP	15.0 ± 8.9 (3.1 to 31.8)	7.5 ± 8.1 (0.1 to 33.3)	0.014*

Data are expressed as median ± standard deviation (percentage or range)

CI, confidence interval; *p < 0.05, **p < 0.01; FPP, functional pelvic plane; APP, anatomical pelvic plane; ACA, anatomical combined anteversion angle; FCA, functional combined anteversion angle

Univariate analysis showed significant differences between the two groups in FPP angle in the supine position, two-stage revision surgery, cemented stem use, functional stem anteversion, FCA in FPP, and the absolute difference between 37.3° and FCA. Only the use of cemented stem (OR, 10.991; 95% CI, 1.064–113.579; *p* = 0.044) and the absolute difference between 37.3° and FCA (OR, 1.146; 95% CI, 1.040–1.263; *p* = 0.006) were identified as significant risk factors for hip dislocation (Table 2).

**Table 2:** Multivariate analysis of the dislocation and non-dislocation groups using binomial logistic regression analysis

	**Odd ratio**	**95% CI**		***p*-value**
		**Lower limit**	**Upper limit**	**(95% CI)**
Two-stage revision	6.425	0.983	42.011	0.052
Cemented stem	10.991	1.064	113.579	0.044*
FPP angle in supine position	0.964	0.964	1.052	0.411
|37.3° − FCA|	1.146	1.040	1.263	0.006**

CI, confidence interval; *p < 0.05, **p < 0.01; FPP, functional pelvic plane; FCA, functional combined anteversion angle

**Fig. 3:**
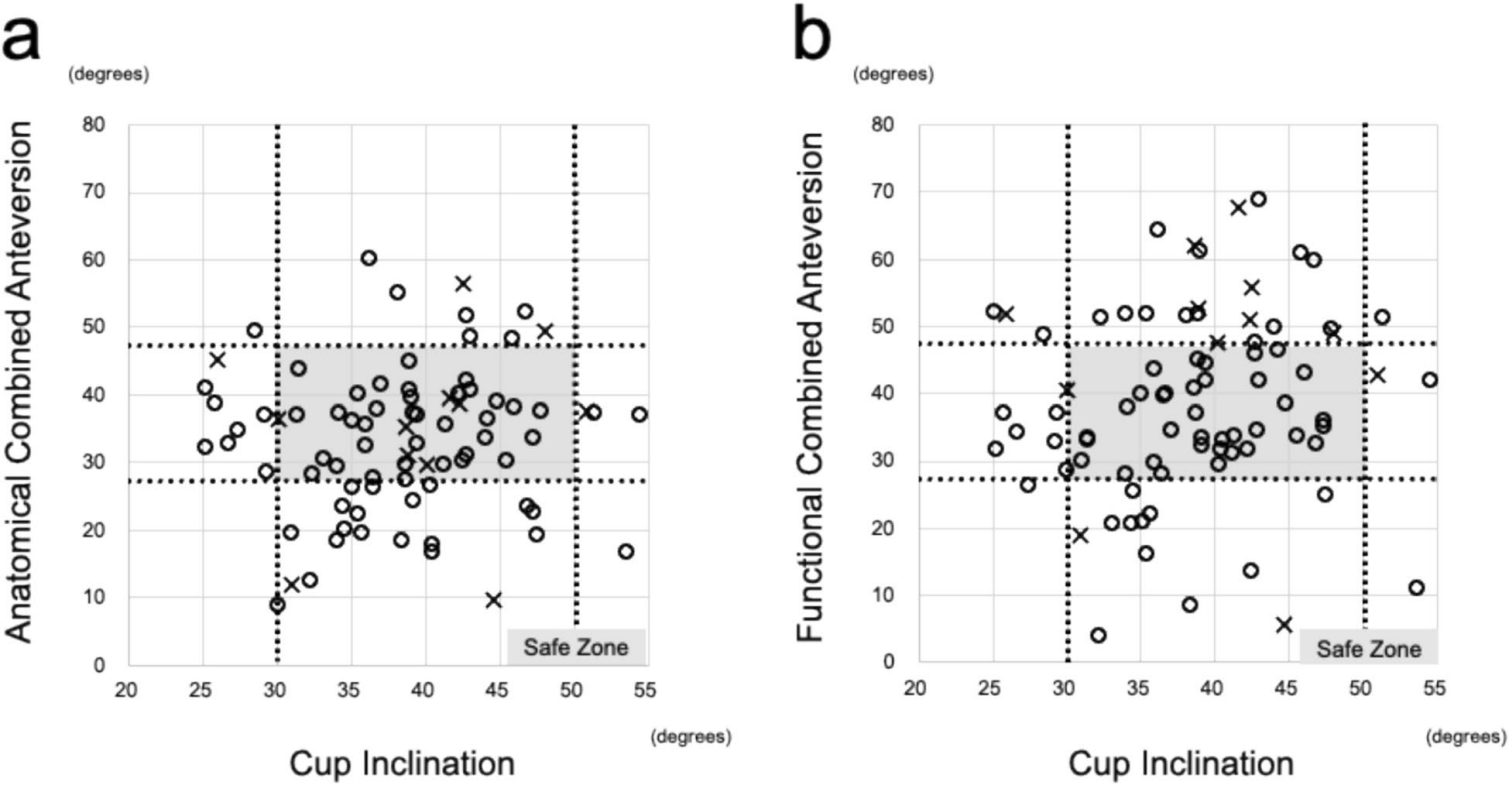
Scatter plots of the anatomical and functional combined anteversion and cup inclination. The horizontal axis is set as cup inclination in the supine functional pelvic plane. The vertical axis is (**a**) the anatomical or (**b**) the functional combined anteversion angle in the functional pelvic plane. Circles indicate the non-dislocation groups, and crosses indicate the dislocation groups. Safe zone: combined anteversion 37.3 ± 10° and cup inclination 40 ± 10°

In the scatter plots of all cases, 5 of 12 hips in the dislocation group for ACA were within the safe zone, a hip was almost directly above the borderline, and 6 hips deviated from the safe zone (Fig. 3). Meanwhile, 2 of 12 hips with FCA were located directly above the borderline, and all the other 10 hips deviated from the safe zone. Focusing on each case of dislocation, 10 patients in whom FCA was higher than ACA due to external femoral rotation had anterior dislocation (Table 3). In contrast, two patients with lower FCA because of internal femoral rotation had posterior dislocation. Additionally, 3 of the 12 patients underwent an additional revision THA because the dislocation could not be controlled with a hip brace alone.

**Table 3:** Detailed data of the 12 patients in the dislocation group

No	Diagnosis	Direction of dislocation	Postoperative days until dislocation (days)	Combined anteversion angle in FPP (°)			Outcome
				**Anatomical**	**Functional**	**Difference**	
1	PJI	Anterior	7	36.5	40.4	3.9	Hip brace
2	PJI	Anterior	26	37.6	42.9	5.3	Stem re-revision
3	Aseptic	Anterior	520	31.1	52.8	21.7	Hip brace
4	PJI	Anterior	42	45.1	51.8	6.7	Hip brace
5	PJI	Anterior	24	49.3	49.0	− 0.3	Stem re-revision
6	PJI	Anterior	7	39.6	67.6	28.0	Cup re-revision
7	Aseptic	Anterior	114	38.6	50.9	12.3	Hip brace
8	Aseptic	Posterior	8	9.7	5.5	− 4.2	Hip brace
9	PJI	Anterior	42	29.7	47.5	17.9	Hip brace
10	PJI	Anterior	662	56.5	55.8	− 0.7	Hip brace
11	Aseptic	Anterior	17	35.4	62.0	26.6	Hip brace
12	PJI	Posterior	26	11.9	18.9	7.0	Hip brace
Mean ± standard deviation			125 ± 212	35.1 ± 13.6	45.4 ± 17.4	10.3 ± 10.9	

In each case, the outcomes were treated after dislocation. Three patients underwent additional re-revision surgery, whereas the remaining patients were treated with hip braces only. Difference was calculated as FCA − ACA, and positive values indicated FCA greater than ACA

FPP, functional pelvic plane; PJI, periprosthetic joint infection; hip brace, hip abduction brace; FCA, functional combined anteversion angle; ACA, anatomical combined anteversion angle

## Discussion

We found that the FCA was higher and deviated more from Widmer’s ideal value in the dislocation group than in the non-dislocation group [9]. In some dislocation cases, FCA deviated from the safe zone when post-operative femoral rotation was considered, although ACA was in the safe zone. No significant difference in the anatomical implant placement was observed between the dislocation and non-dislocation groups. This suggests both the anatomical component placement angle and *F*CA, including post-operative femoral rotation and posterior pelvic tilt, may influence dislocation risk. Previous studies mainly focused on the ACA or conventional combined anteversion using two-dimensional measurements. In contrast, this study used CT-based three-dimensional modeling to assess FCA, which may better represent functional alignment and instability risk [8, 9]. As shown in Table 3, anterior dislocation tended to occur when FCA exceeded Widmer’s ideal combined anteversion of 37.3°, whereas posterior dislocation occurred when FCA was lower than 37.3° [9]. Muscle imbalance, particularly weakness of the gluteus medius and iliopsoas, may contribute to external femoral rotation after revision THA. We considered that anatomical planning within the safe zone would not be reliable in terms of post-operative dislocation, especially in cases with strong pre-operative external femoral rotation due to damaged muscles or soft tissues. When soft tissue damage or bone loss occurs because of instability or infection, identifying landmarks during revision THA is often difficult, possibly resulting in greater anteversion angles of the cup than those planned. The use of navigation during revision THA is important [10, 16, 17] because only mechanical guiding or free guiding techniques are insufficient for accurate placement of acetabular components [18, 19]. Although the use of dual-mobility cups was not significantly associated with a reduction in dislocation rates in this study, previous reports have demonstrated that they are effective in reducing the risk of post-operative dislocation following revision total hip arthroplasty [20]. Therefore, the navigation system and dual-mobility cups may function as complementary strategies to reduce the risk of dislocation, particularly in severe revision cases. Furthermore, if the femur is assessed as being externally rotated post-operatively, rehabilitation may require limiting external rotation.

Two-stage revision surgery was performed in 75% of patients (9 of 12 cases) in the dislocation group compared to 31.4% in the non-dislocation group. This is due to the reduced muscle strength during the waiting period for surgery and reduced lower-limb tension caused by soft tissue damage from multiple surgeries. A meta-analysis by Guo et al. suggested that older age, smaller head diameter, history of instability, and number of revision THAs performed were risk factors for dislocation after revision THA [2]. Muscle weakness in the psoas major, iliopsoas, and gluteus medius may result in external femoral rotation because these muscles are involved in hip stability [15]. All except one patient in this study underwent surgery using a lateral approach, preserving the short external rotator musculature. Therefore, we suspected that the deviation of FCA with external femoral rotation from the safe zone would result in hip instability. In addition, the femoral external rotation angle tended to be higher in the dislocation group just before two-stage revision surgery, although the difference was not statistically significant. This suggested that soft tissue damage or muscle weakness might cause the femur to remain externally rotated even after surgery.

The number of patients with cemented stems was higher in the dislocation group than in the non-dislocation group, and our multivariate analysis indicated that the use of a cemented stem was also associated with an increased risk of dislocation. This may be because cemented stems are often used in cases with severe femoral bone loss or soft tissue damage at the femoral side. These conditions may indicate more complex surgical situations with reduced femoral support and soft tissue tension disorders, which may increase the instability of implants. Therefore, the observed association may represent the underlying severity of femoral defect and soft tissue damage rather than a direct mechanical disadvantage of cement fixation itself. A previous systematic review indicated no significant difference between cemented and cementless stems in revision THA in terms of complication rates, including dislocations [21]. Cement selection in revision THA is largely determined by femoral bone quality and surgical complexity, which may confuse the relationship between fixation types and dislocation risk.

Babisch et al. reported that a 1° change in posterior pelvic tilt resulted in a 0.8° change in cup anteversion [22]. Therefore, the FPP angle is also essential in preventing instability related to the CA. FPP angle measurement by three-dimensional modelling in all cases also showed that the FPP angle was lower in the dislocation group than in the non-dislocation group. After primary THA, some patients gradually develop progressive posterior pelvic tilt [23, 24], which may be one reason for the dislocation risk after revision surgery because of a greater CA.

The decision to perform re-revision surgery depended on the attending physician, and three patients (25.0%) with peri-prosthetic joint infection underwent reoperation. In the other nine cases, the use of a hip brace was able to prevent redislocation. A hip abduction brace can control the hip range of motion in extreme flexion and adduction [25]. Patients with dislocation during revision THA are sometimes required to wear a hip brace for 3 months [26]. However, the optimal duration of brace use after dislocation remains controversial.

This study has four limitations: short-term or medium-term clinical outcomes, insufficient assessment of posterior pelvic tilt and femoral rotation in the standing or sitting position, insufficient assessment of soft tissue tension, and a certain number of excluded cases. Although we determined a post-operative follow-up period of 1 year, two patients in the dislocation group experienced dislocation at > 1 year after revision THA (cases 3 and 10). Some patients were expected to develop dislocations in the future, even if they had never experienced a dislocation to date. Sustained follow-up is essential, especially for patients with deviated FCA, because more patients will possibly experience dislocation in the future. Risk factors for dislocation can be further examined by including an assessment of femoral rotation and posterior pelvic tilt at the time of dislocation. This study was conducted with patients in the supine position on a CT table; therefore, the FPP angle and femoral rotation in the standing or sitting position could not be assessed [27]. External rotation may change during the early post-operative period due to pain, muscle recovery, and gait adaptation. Therefore, the measured rotation may not fully reflect long-term functional rotation or rotational alignment at the time of dislocation. In addition, spinopelvic parameters, including spinopelvic mobility and sagittal alignment, were not evaluated in this study. These parameters have been reported to influence pelvic orientation and functional acetabular component position, particularly during postural changes [24]. Future studies using dynamic imaging or slot-scanning 3D X-ray imaging systems (EOS) may improve functional position assessment [28]. In clinical practice, it is important to avoid damage to soft tissues such as muscles, joint capsules, and ligaments in cases of revision THA with multiple operations. Six cases had undergone re-revision surgery prior to this study. The number of re-revision surgeries and surgical indications was comparable between the two groups. These findings indicated that the need for re-revision was not specific to the dislocation group. Although the angle of implant placement is within the safe zone, patients with soft tissue loss and affected lower-limb shortening may dislocate their hip joints because of low tension. Although accurate intra-operative measurement of lower-limb tension may help reduce instability, reliable evaluation methods have not yet been established. Patients with dislocation tend to be followed up; whereas many patients without dislocation are lost to follow-up, which may result in a high dislocation rate among patients. In addition, the limited number of post-operative dislocations restricted the number of variables that could be included in the multivariate analysis. Therefore, the results of the multivariate analysis should be interpreted as exploratory, and residual confounding could not be completely ruled out. Future research should focus on further assessments in the functional positions and should aim to develop better intra-operative techniques to address soft tissue tension discrepancies to improve the patients’ outcomes and long-term success in revision THA. Accordingly, this study should be regarded as exploratory in nature, and caution is required when extrapolating the findings to broader populations. Future multicenter studies with larger cohorts are needed to validate these findings.

## Conclusions

Deviation of FCA with femoral rotation was associated with post-operative dislocation after revision THA. These findings suggested that functional pelvic alignment and femoral rotation might be important for reducing the risk of dislocation in revision THA.

## Supplementary Information

Supplementary Material 1.

## Data Availability

The datasets used and analyzed in this study are available upon reasonable request by contacting the corresponding author.

## Abbreviations

THA: Total hip arthroplasty
CA: Combined anteversion
ACA: Anatomical combined anteversion
FCA: Functional combined anteversion
CT: Computed tomography
BMI: Body mass index
FPP: Functional pelvic plane
APP: Anatomical pelvic plane
OR: Odds ratio
CI: Confidence interval
ICC: Intraclass correlation coefficients
EOS: Slot-scanning 3D X-ray imaging system
