# Supplementary material for: Association between functional combined anteversion and dislocation after revision total hip arthroplasty
Source: Arthroplasty. 2026 Apr 10;8:26. doi: 10.1186/s42836-026-00383-w (PMC13067479; doi:10.1186/s42836-026-00383-w)
Supplement: Supplementary file 1 — Supplementary Material 1. [file 42836_2026_383_MOESM1_ESM.docx]

***Supplementary Materials***

**Functional pelvic plane**

The functional pelvic plane (FPP) angle in the supine position for imaging on a computed tomography (CT) table was calculated using the software. We measured and defined the angle between the plane of the table, the three points of the bilateral anterior superior iliac spines, and the anterior surface of the pubic symphysis in the supine position as the FPP angle. Lateral flexion of the pelvis was simultaneously corrected at the same time.

**Femoral rotation angle**

The effect of pelvic rotation was corrected by measuring the bilateral anterior superior iliac spine tilt in the horizontal section of the CT images, similar to the method used for stem anteversion. The femoral rotation angle was defined as the angle between the straight line connecting the contact point of the posterior condyle on CT images and the line parallel to the table on the affected side. The direction of the external rotation was assumed to be positive.

**Stem anteversion**

Anatomical stem anteversion was defined as the angle between the stem neckline and posterior condylar axis in the axial plane (Supplementary **Fig. S1**). Functional stem anteversion was defined as the angle of the sum of the anatomical stem anteversion and external femoral rotation. In cases where the femur was internally rotated, the functional stem anteversion angle was defined as the anatomical stem anteversion angle minus the femoral rotation angle. The effect of pelvic rotation was corrected by calculating the bilateral anterior-superior iliac spine tilt in the horizontal section of the CT image.

**Cup anteversion and cup inclination**

We measured the anteversion and inclination of the cup by fitting the same-sized component data in each of the three directions to a three-dimensional pelvic model and components using CT images obtained within 2 weeks after surgery (Fig. 2a, b). Cementless or metal-cemented cups were visible on CT images, whereas polyethylene cemented cups were not visible. The anteversion and inclination of the cemented polyethylene cups were measured by conforming the same-sized radiopaque wires (X3 Rimfit, Stryker, Portage, MI, USA) to the full circumference of the cup models. All cup angles were measured with the patient in the supine position by the software. For cup angle measurement, the anatomical pelvic plane (APP) and FPP were used as the reference planes, and lateral flexion and rotation of the pelvis were corrected to 0°.

**Leg length difference**

Leg length was defined as the distance from the anterior superior iliac spine to the center of the internal and external condyles of the knee, with the lower limb in the same position, bilaterally on the three-dimensional pelvic model. Leg length differences were measured at the pelvic angle of each APP and FPP. The leg length of each patient was compared bilaterally and considered positive when the affected side was longer.

**Offset difference**

Pelvic and femoral offsets and their total offsets were measured. A stem of the same size as the surgically placed one was adapted to each postoperative CT image (Fig. 2c). For the pelvic offset, the distance in the anterior view from the pubic symphysis and sacral median line to the center of the femoral head was measured with the three-dimensional bone model. For the femoral offset, the postoperative perpendicular distance from the center of the head to the femoral shaft extension was measured on the three-dimensional distance. The femoral shaft extension was defined as the line connecting the third and fifth centers from the proximal side of the femoral model, divided into 14 sections. The total offset was the length of the sum of these offsets. The offset difference was considered positive when the affected side was longer.


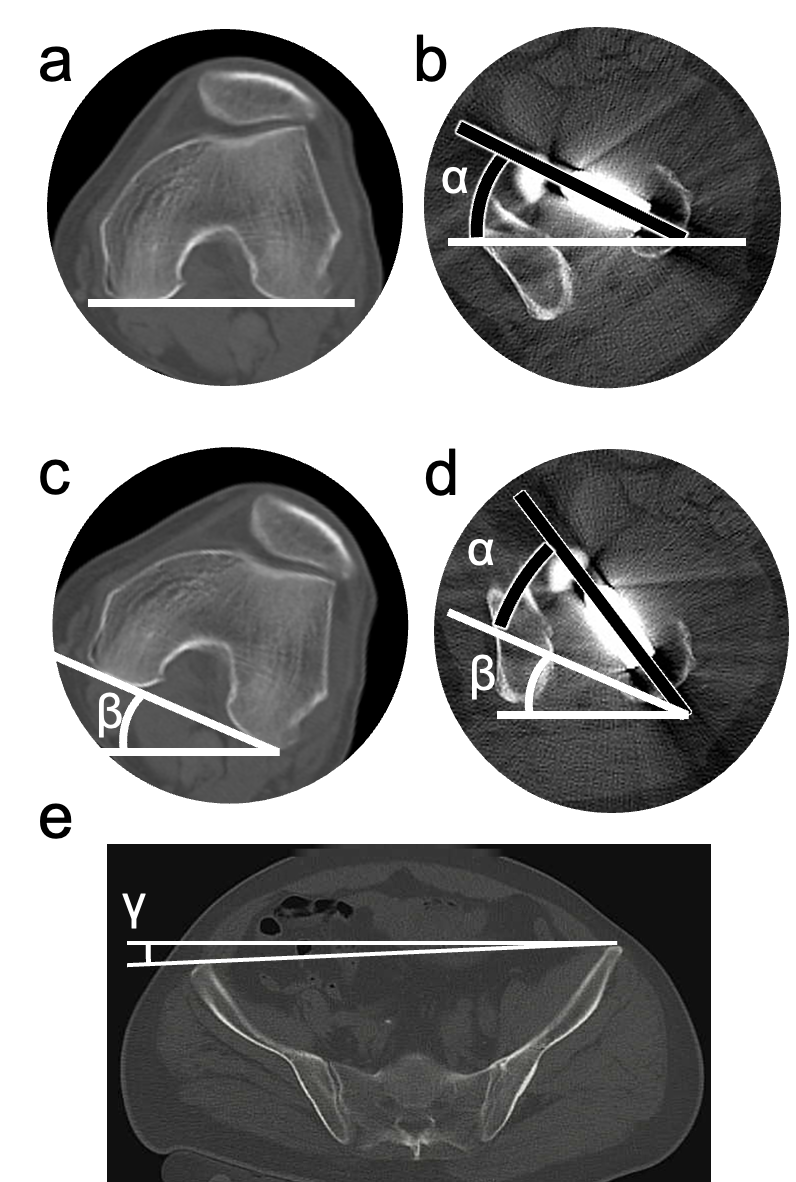


**Fig. S1.** Method of measuring the femoral rotation and stem anteversion angles using computed tomography images at 1 month before and within 2 weeks after surgery. (**a, b**) The anatomical stem anteversion angle is defined as α when the femoral rotation is 0°. (**c, d**) The functional stem anteversion angle is defined as α + β when β is the external femoral rotation angle. (**e**) When measuring the anatomical or functional anteversion angle or femoral rotation, the pelvic rotation angle (γ) is corrected to 0°.
